# New South Wales Asylum

**Published:** 1892-09-10

**Authors:** 


### THE STORY OF THE INSANE FROM YEAR TO YEAR.

(Fourth Series.)

### NEW SOUTH WALES ASYLUM.

From the report of the talented Inspector-General of Asylums, Dr. Manning, we learn that the insane population of New South Wales rose during the year from 2,974 to 3,102, showing an increase of 128. On page two of his report, Dr. Manning gives us a most interesting table, wherein he contrasts the relative frequency of insanity in England and in New South Wales. It would seem that in the year 1871 England had one lunatic to every 394 sane persons and New South Wales had one to 374. In the year 1889 the proportion had risen in England to one in 341, whereas in New South Wales it had slightly fallen to one in 377; and it should be borne in mind that this decrease has been in spite of many insane persons landed on these shores; the bulk of these lunatics being no doubt from England. What is the cause of this comparative immunity? It can hardly be the percentage of recoveries, for that stands at 42·73 on the admissions, which is only 2 per cent. above our English recovery rate; and this point would be more than balanced by the low death rate which stands at 6·75. We find that the admissions numbered 685: the recoveries to 257, and the deaths to 193. Hereditary influence was the ascertained cause in 53 of the admissions, and drink in 71; and, sad as that tale is, it would have to be greatly added to if the causes in 241 cases entered as "unknown" could be discovered. Consumption was the cause of death in 25 cases; this scourge of civilized man seems to follow him everywhere. Victoria, South Australia, Queensland, Tasmania, and New Zealand have Acts forbidding the importation of insane and otherwise helpless and dependent persons, the colony of New South Wales follows the bad example of the Mother Country and allows the improvident and helpless of all countries to be a burden to her. Dr. Manning wisely draws attention to this fact so far as it affects New South Wales, and asks why an Act of Parliament of one or two sections should not be passed, placing the colony in the same position as its neighbours. Alas! we have often asked the same question as regards England; but the hope of remedy now lies almost dead within us. Thousands and thousands of Irish lunatics and a few hundreds, at least, of Scotch, crowd our county asylums, and we make no effort to get rid of them. No member of Parliament introduces a short Bill of two clauses for us, and so we grumble and—pay our taxes. It does not seem necessary to review the various reports of the asylums, but we may finish this notice by saying that the Gladesville Asylum contains 795 lunatics; that at Paramatta, 1,002; Callan Park, 772; Newcastle, 245; and the Criminal Asylum, 54.

